# Evolving Horizons in Temporomandibular Joint Total Replacement: A Comprehensive Narrative Review

**DOI:** 10.7759/cureus.95806

**Published:** 2025-10-31

**Authors:** Yellanti Doondi Dinesh Nag, Ravada Kinneresh, Morthala Bala Venkateswara Reddy, Mudunoori Mrudulika, Anjan K Karri, Balajee G Bandi, Eswaravaka Narahari, Seema Gupta

**Affiliations:** 1 Department of Oral and Maxillofacial Surgery, Clove Dental Clinic, Hyderabad, IND; 2 Department of Public Health Dentistry, Great Eastern Medical School and Hospital, Srikakulam, IND; 3 Department of Dentistry, 247 King Dental, Waterloo, CAN; 4 Department of Community Medicine, Sri Venkateswara Institute of Medical Sciences, Tirupati, IND; 5 Department of Orthodontics, Government Dental College and Hospital, Kadapa, IND; 6 Department of Orthodontics, Kothiwal Dental College and Research Centre, Moradabad, IND

**Keywords:** end-stage, replacement, review, surgery, temporomandibular joint

## Abstract

Total temporomandibular joint (TMJ) replacement has become a vital solution for managing end-stage TMJ disorders and significantly enhancing mandibular function and patient quality of life. This narrative review explores the historical developments, clinical objectives, biomaterials, devices, surgical techniques, preoperative preparation, complications, management approaches, and recent innovations in TMJ total joint replacement (TJR). The review draws from a literature search conducted across PubMed, Scopus, Web of Science, and Google Scholar from database inception to August 10, 2025, using terms like "total temporomandibular joint replacement," "TMJ TJR," and "alloplastic TMJ prosthesis," focusing on English-language peer-reviewed articles, reviews, and clinical studies. Current practice leans toward alloplastic reconstruction, utilizing titanium alloys for osseointegration and ultra-high molecular weight polyethylene (UHMWPE) for low-friction surfaces, supported by custom designs via computer-aided design and manufacturing (CAD/CAM). Surgical approaches, including preauricular, retromandibular, and rhytidectomy techniques, have been refined to protect critical structures, such as the facial nerve and internal maxillary artery. Preoperative preparation involves patient education on risks, such as infection and nerve injury; advanced imaging with computed tomography (CT) and 3D reconstructions; and strict aseptic measures. Complications, such as heterotopic bone formation and dislocations, are managed with excision, fat grafting, or surgical redesign, while nerve injuries and synovial issues require targeted interventions. Recent advances have highlighted bioengineered solutions, including bioresorbable composites and nanomaterials, that are promising for merging alloplastic and biological benefits. Currently, these developments, underpinned by ongoing research, position TMJ TJR as a cornerstone of maxillofacial surgery, with the potential to reduce donor morbidity and improve long-term outcomes, although challenges such as infection rates and material hypersensitivity persist, necessitating continued refinement.

## Introduction and background

End-stage joint disease describes a joint that is structurally damaged by disease or injury, resulting in profound functional impairment. Similar to other joints, the temporomandibular joint (TMJ) is susceptible to end-stage pathologies stemming from developmental anomalies, neoplasia, trauma, arthritic conditions, unsuccessful prior surgeries, or fibrous/bony ankylosis [1]. End-stage TMJ disease results in discomfort, limited oral aperture, joint degeneration, and significantly diminished quality of life. Moreover, both osseous resorption and growth inhibition may result in a retrognathic position of the mandible, consequently precipitating obstructive sleep apnea (OSA) [2]. Irrespective of the cause, reconstructive surgery seeks to enhance mandibular function and structure, alleviate suffering and disability, curb excessive treatment and expenses, and avert additional morbidity [1,2].

TMJ reconstruction presents distinctive challenges compared to reconstruction of other joints, given its pivotal involvement in the stomatognathic, upper respiratory, and digestive systems. It functions as a secondary mandibular growth center during prepubertal years and is indispensable for mastication, speech, airway maintenance, and swallowing in children and adults. The available modalities for total TMJ reconstruction include autogenous bone grafting, alloplastic implants, and transport distraction osteogenesis, with bioengineered alternatives being explored [1-4].

Alloplastic reconstruction has emerged as the primary treatment for end-stage TMJ disorders and was originally applied in cases of trauma, advanced joint disease, repeated failed surgeries, and post-ablative reconstructions. Growing evidence supports its use in adult TMJ ankylosis and end-stage disease. Alloplastic prostheses effectively achieve reconstruction objectives, although evidence of their application in growing patients is limited. The implantation site poses significant challenges involving biochemical, biological, and biomechanical elements within evolving living tissues that impact prosthesis durability [2,5].

The initial TMJ total joint replacement (TJR) occurred in the 1970s; however, its early adoption was limited. Failures with materials such as Proplast and Silastic in the 1990s caused setbacks in North America, whereas Japan emphasized autogenous reconstructions. Innovations such as the Sonnenburg prosthesis and FDA approvals for Biomet (1995), TMJ Concepts (1999), and NEXUS CMF (2001) have advanced the field. Full U.S. approval for Biomet followed in 2005, and Japan approved TJR in 2019, facilitating clinical implementation amid demanding high-risk procedures requiring judicious patient selection [6].

The aim of this narrative review was to synthesize existing knowledge on total TMJ TJR, covering history, goals, biomaterials, devices, surgical approaches, preparation and surgery, complications, management, and recent advances, based on pertinent literature to inform clinical decision-making.

## Review

Search methodology

This narrative review employed a structured methodology inspired by the Preferred Reporting Items for Systematic Reviews and Meta-Analyses (PRISMA) guidelines to promote transparency and thoroughness while accommodating the narrative style for expansive synthesis. Literature searches were performed across databases, such as PubMed, Scopus, Web of Science, and Google Scholar, from database inception to August 2025. Search terms included "total temporomandibular joint replacement," "TMJ TJR," "alloplastic TMJ prosthesis," and "end-stage TMJ disease," paired with specifics such as "history," "goals," "biomaterials," "devices," "approaches," "surgery," "complications," "management," and "advances." Boolean operators (AND/OR) were used, with English-language, peer-reviewed articles, reviews, clinical trials, and case series prioritized. Non-human studies, grey literature, and conference abstracts were excluded.

**Figure 1:**
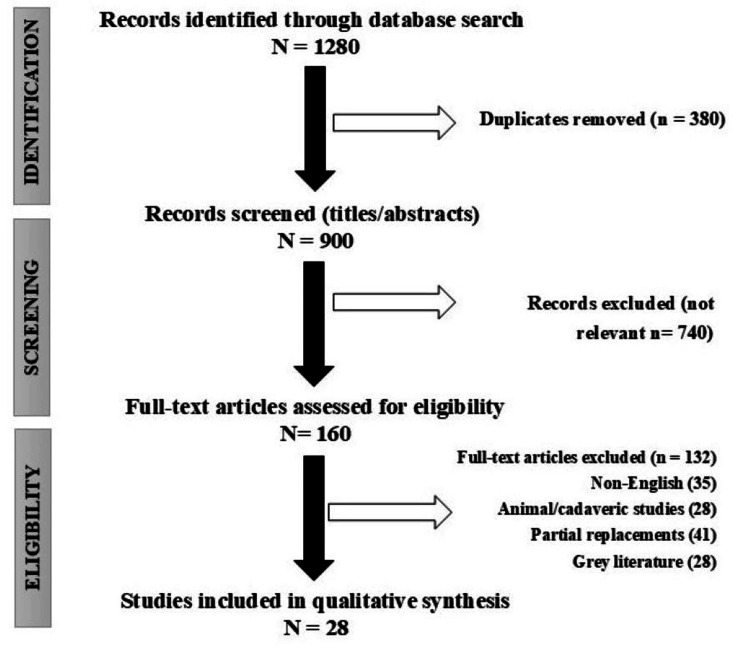
Preferred Reporting Items for Systematic Reviews and Meta-Analyses (PRISMA) flowchart outlining the study selection process

The initial search retrieved 1,280 records. Duplicates were eliminated (n = 380), yielding 900 titles and abstracts that were screened for TMJ TJR relevance. Full texts (n = 160) were evaluated, excluding those on partial replacements or unrelated subjects (n = 132). Twenty-eight articles were selected for qualitative synthesis, with a focus on impactful reviews and outcome studies. No quantitative meta-analysis has been conducted because of the narrative focus. This method ensures balanced evidence from various sources, including historical perspectives and contemporary innovations (Figure 1).

History of TMJ TJR

The evolution of TMJ TJR reflects a long journey from rudimentary interventions to sophisticated alloplastic solutions, driven by the need to address severe joint dysfunction [7]. Historical records indicate that TMJ disorders have been recognized since ancient times, with early references in Egyptian hieroglyphics around 5000 BC describing ankylosis and dislocation. In 1840, attempts to mobilize the TMJ using a wood block after gap creation at the condyle neck represented an early interpositional approach, while the use of ivory prostheses for the TMJ and hip joints led to the introduction of alloplastic materials stabilized with cement [6,8].

The early 20^th^ century saw a shift toward autogenous tissues for reconstruction. The fourth metatarsal was used for condylar replacement. Later, gold leaves were introduced into the joint fossa, and autogenous metatarsal heads were utilized by many surgeons. Interpositional materials such as tantalum foil were employed, followed by the introduction of stainless steel for fossa replacement, and the 1960s brought cobalt-chromium alloys such as Vitallium. Polymers, including silicone and polytetrafluoroethylene, are used as disc replacements. Later, non-vascularized metatarsophalangeal joints with vascularized variants were introduced to treat facial deformities. Vascularized rib and iliac crest grafts have also gained traction [6-8].

Despite these advances, alloplastic TMJ TJR has significant challenges and setbacks. The 1970s marked the first TJR procedure, but materials such as Proplast-Teflon and Silastic led to giant cell reactions and failures in the 1980s-1990s, prompting recalls in North America [8]. In contrast, Japan has prioritized autogenous fascial or fat grafting to avoid problematic devices [7]. The Sonnenburg prosthesis, which features a titanium condyle and polyethylene fossa, emerged in the 1980s as a viable alternative. Regulatory milestones in the 1990s included FDA trial approval for Biomet in 1995, TMJ Concepts in 1999, NEXUS CMF in 2001, and full Biomet approval in 2005. Japan's 2019 approval enabled clinical use, although its high-risk nature demands careful patient selection. These developments underscore improved biomaterials and designs that enhance longevity and efficacy in end-stage cases. Recent reviews have highlighted how historical failures inform current protocols, emphasizing biocompatibility and long-term outcomes (Table 1) [6-9]. 

**Table 1:** Functional outcomes and long-term success of major TMJ replacement systems TMJ: temporomandibular joint; CAD/CAM: computer-aided design and manufacturing

Study/Device	Sample Size (n)	Follow-up (Years)	Mean Interincisal Opening (mm)	Pain Reduction (%)	Complication Rate (%)	Reference
Biomet Total TMJ	120	10	30–35	70–80	15	Westermark , 2010 [10]
TMJ Concepts Custom	110	8	28–32	80–85	10–12	Almeida et al., 2025 [11]
NEXUS CMF	60	5	27–30	65–70	18	Baecher et al., 2025 [9]
Custom CAD/CAM	40	4	33	85–90	<10	Irshad et al., 2025 [12]
Autogenous Rib Graft	55	6	25–28	60–65	25	Hawkins et al., 2020 [4]

Goals of TMJ reconstruction

The objectives of TMJ reconstruction, whether through alloplastic, autogenous, or other methods, are multifaceted and aim at restoring optimal function while minimizing patient burden. Primarily, these goals include improving the mandibular form and function, reducing pain and disability, containing costs, and preventing further morbidity. In patients with severe architectural distortions from end-stage disease, total reconstruction is essential; however, achieving premorbid normality is often unrealistic because of the joint's complexity and surrounding muscle dynamics [3]. For multiple operated patients with anatomical alterations and chronic neuropathic pain, the focus shifts to objective restoration of mandibular function and form, with pain relief as a secondary benefit.

Alloplastic TMJ TJR is particularly indicated for conditions such as inflammatory arthritis unresponsive to conservative treatment, recurrent ankylosis, failed grafts, and loss of vertical height or occlusion from resorption, trauma, or lesions. In inflammatory arthritis, in which synovial destruction persists, complete synovectomy is impractical, and the orthopedic literature supports alloplastic replacement for predictable results [13]. Dissatisfaction with autogenous costochondral grafting in highly inflammatory or ankylotic cases has propelled TJR adoption, backed by data showing superior stability and reduced reoperation rates [1,7]. For re-ankylosis, placing autogenous bone in reactive environments is counterproductive, mirroring orthopedic preferences for alloplastics [12].

In pediatric or growing patients, autogenous grafts, such as costochondral grafts, are traditional but unpredictable, often resulting in ankylosis due to fixation challenges or non-compliance. Alloplastic TJR lacks growth potential but may be suitable for severe cases, drawing from orthopedic experiences to improve quality of life. Failed tissue grafts in scarred avascular beds highlight autogenous limitations, as thick scar tissue impedes vascular penetration, leading to resorption. Literature indicates that alloplastics are more reliable in such scenarios, thus avoiding donor morbidity [5,14].

Previous alloplastic failures, involving osteolysis and foreign body reactions, complicate subsequent reconstructions; however, long-term studies have shown functional success despite subjective variability from materials such as Proplast-Teflon [14,15]. Addressing posterior mandibular height loss from pathologies requires TMJ-focused reconstruction rather than osteotomies to correct deformities, such as open bites. Relative contraindications include young age, unrealistic expectations, uncontrolled systemic diseases, active infections, and allergies. Overall, the TMJ TJR aligns with these goals by providing immediate stability and durability when appropriately selected, balancing the risks and benefits for optimal patient outcomes (Table 2).

**Table 2:** Comparative overview of TMJ replacement systems TMJ: temporomandibular joint; Co–Cr–Mo: cobalt–chromium–molybdenum; Ti–6Al–4V: titanium alloy containing 6% aluminum and 4% vanadium; CAD/CAM: computer-aided design and manufacturing; HA: hyaluronic acid

Material/Device	Manufacturer	Composition	Advantages	Limitations	Longevity (years)	Supporting References
Biomet Stock Prosthesis	Zimmer Biomet, USA	Co–Cr–Mo condyle with UHMWPE fossa	Robust, high wear resistance, modular sizing	Limited customization, allergic potential	10–15	Kanatsios et al., 2018 [16]; Zou et al., 2018 [17]
TMJ Concepts Custom Prosthesis	TMJ Concepts, USA	Ti–6Al–4V alloy with UHMWPE fossa	Patient-specific CAD/CAM design, excellent stability	Cost, fabrication delay	15+	De Meurechy et al., 2020 [18]; Almeida et al., 2025 [18]
NEXUS CMF	NEXUS CMF Ltd., UK	Titanium alloy	Lightweight, adaptable fit	Limited long-term outcome data	10–12	Baecher et al., 2025 [9]
Autogenous Costochondral Graft	—	Rib cartilage and bone	Growth potential in young patients	Resorption, re-ankylosis risk	Variable	Hawkins et al., 2020 [4]
Nanocomposite/Bioresorbable Scaffold	Experimental	HA-polymer or composite	Biocompatible, osteoconductive	Preclinical stage	—	Velasco et al., 2015 [19]; Rezazadeh et al., 2024 [20]

TJR biomaterials

The selection of biomaterials for TMJ TJR is critical for ensuring safety, efficacy, and longevity, as past failures with synthetic materials underscore the need for continuous improvement to meet clinical and regulatory standards. The early replacements used polymethylmethacrylate and metal-on-metal articulations, which caused excessive wear, inflammation, and device failure. Cobalt-chromium-molybdenum alloys are preferred owing to their strength, polishability, and wear resistance; however, concerns over particle debris and tissue reactions persist, prompting the exploration of ceramics and ultra-high molecular weight polyethylene (UHMWPE) to minimize these issues [8].

Implant surface properties play a key role in biocompatibility and integration, with cleanliness, energy, and chemical composition influencing the tissue response. Techniques such as electron spectroscopy (e.g., X-ray photoelectron spectroscopy (XPS)) and surface energy measurements optimize these attributes, whereas sterilization methods must preserve them. Advancements in computer-aided design and manufacturing (CAD/CAM) have facilitated custom devices that are likely to dominate in the near term [12]. Wear-resistant UHMWPE has replaced older polymers, and innovations in ceramics and lubricants have aimed to further enhance their performance [21].

Specifically, for TMJ TJR, biomaterials must withstand corrosion, provide strength, and exhibit low wear while being biocompatible. Titanium alloys, such as titanium alloy containing 6% aluminum and 4% Vanadium (Ti-6Al-4V), are commonly used for condylar and fossa components owing to their osseointegration properties. UHMWPE offers low-friction articular surfaces and reduces the amount of debris. Cobalt-chromium-molybdenum provides hardness but carries a hypersensitivity risk. Surface coatings such as hydroxyapatite promote better tissue integration, and biocompatibility testing ensures minimal inflammatory responses. Recent studies have demonstrated reduced particle generation in modern generations compared to their predecessors. Bioresorbable composites and nanomaterials are promising for regenerative applications, potentially bridging alloplastic and biological reconstructions [7,22,23]. The integration of bioinformatics and computational modeling positions TMJ TJR at the frontier of personalized reconstructive surgery.

TMJ TJR devices

TMJ TJR devices are broadly classified into stock (prefabricated) and custom (patient-specific) types, each offering distinct advantages in different clinical scenarios. Stock prostheses, developed in the 1990s without initial rigorous pre-market approval, include models such as the Morgan, Christensen, Kent-Vitek, Osteomed, and Delrin-Timesh condylar designs. The Biomet (now Zimmer Biomet) total TMJ implant represents a key stock system, assuming that skeletally immature patients benefit from autogenous options or distraction, while mature patients with standard indications achieve safe outcomes in non-complex cases [18]. However, extensive reconstructions often require custom designs fabricated via CAD/CAM from 3D computed tomography (CT) scans for a precise fit.

Indications for alloplastic devices include late-stage degenerative disease, recurrent ankylosis, irreparable condylar fractures, revisions of failed alloplastic or autogenous reconstructions, avascular necrosis, neoplasia requiring resection, and congenital disorders, such as hemifacial microsomia. Contraindications include allergies to prosthetic materials, chronic infections, systemic conditions that increase infection risk, and skeletal immaturity [13]. The Biomet stock prosthesis features a UHMWPE (ArCom®) fossa of three sizes with variable zygomatic flanges for screw fixation, gamma-radiated for crosslinking and reduced wear, and stabilized by modifying the articular eminence for tripod support. The mandibular component uses cobalt-chrome alloy (ASTM F799) with plasma-sprayed titanium on the ramal plate, available in 45-55 mm lengths and narrow or standard designs [16].

Stock devices offer flexibility in fit, immediate availability for urgent cases such as trauma or tumors, and lower costs, but require surgeon expertise to handle variability and limit anterior-inferior movements. Custom devices provide superior stability in distorted anatomies and accommodate greater excursions, although they may require two-stage procedures, take eight to 12 weeks to fabricate, offer less intraoperative flexibility, and incur higher costs. Overall, stock TJR systems, comprising UHMWPE fossae and cobalt-chrome mandibular components, prioritize durability while mitigating adverse reactions, with ongoing comparisons favoring custom for complex ankylosis but stock for standard applications (Table 3) [17,18].

**Table 3:** Evolution of TMJ TJR TMJ: temporomandibular joint; TJR: total joint replacement; CAD/CAM: computer-aided design and manufacturing; Ti–6Al–4V: titanium alloy containing 6% aluminum and 4% vanadium; UHMWPE: ultra-high molecular weight polyethylene

Year/Period	Milestone	Material/Device	Significance	Supporting References
1840s	First interpositional attempt using a wooden block after condylar gap creation	Wood/Ivory	Early mechanical approach to gap arthroplasty	Driemel et al., 2009 [6]
1920s	Gillies introduces the metatarsal graft for condylar reconstruction	Autogenous bone graft	Early biological replacement	Liu et al., 2025 [7]
1960s	Stainless steel and Vitallium prostheses were introduced	Metal alloys	Improved durability, reduced corrosion	Driemel et al., 2009 [6]
1970s	First total TMJ replacement was performed	Proplast–Teflon	Marked the beginning of the alloplastic TMJ era	Mercuri, 2019 [8]
1980s–1990s	Material failures and regulatory recalls	Silicone, Proplast	Shift toward biocompatible alloys	Mercuri, 2019 [8]
1995–2001	Biomet, TMJ Concepts, and NEXUS CMF trials approved	Titanium–UHMWPE	FDA-approved standardized systems	Yadav et al., 2021 [10]
2005	Full FDA approval for the Biomet system	Ti-6Al-4V, UHMWPE	Clinical benchmark	Westermark, 2010 [10]
2010s	CAD/CAM prostheses introduced	Additive titanium	Custom design revolution	Irshad et al., 2025 [12]; Lee et al., 2018 [24]
2020s	Nanocomposites and bioengineered scaffolds are emerging	Hybrid biomaterials	Transition toward regenerative replacements	Baecher et al., 2025 [9]; Acri et al., 2019 [25]

Approaches to TMJ

Accurate diagnosis and meticulous treatment planning are essential for successful TMJ surgery, with the choice of approach directly affecting access, safety, and outcomes. Surgeons must be proficient in various techniques to address the joint's proximity to critical structures, including the facial and auriculotemporal nerves, superficial temporal vessels, middle temporal artery, and the parotid gland. Approaches have evolved to minimize risks, categorized as extraoral (preauricular, endaural, postauricular, retromandibular, submandibular, and intraoral), and designed for specific indications such as fracture reduction, disc surgery, or total replacement.

The submandibular approach involves a 4-5 cm incision of two fingerbreadths below the mandibular border, dissecting the platysma and deep cervical fascia to expose the marginal facial nerve. It provides access but risks nerve damage from retraction and is distant from the fracture site [26]. The retromandibular approach uses a 4 cm incision behind the posterior ramus, dissecting anteromedially to the mandible while navigating the marginal/cervical facial nerves and retromandibular vessels, followed by an incision of the periosteum [27].

Preauricular or auricular approaches are ideal for upper condylar processes, articular disc fractures, intracapsular reductions, and soft tissue procedures; however, ramus access for miniplates is limited. The Al-Kayat & Bramley variant uses a question mark-shaped incision extending into the hairline for better aesthetics and access [28]. The rhytidectomy approach, originally for facelifts, offers less visible scarring and exposes condylar neck fractures via a 1.5-2 cm incision above the zygomatic arch, extending downward in the preauricular fold and posteriorly below the earlobe [29]. Skin flaps are raised subcutaneously and widely undermined. When selected judiciously, these approaches optimize the TMJ TJR by reducing complications and ensuring precise prosthesis placement.

TJR preparation and surgery

Preoperative preparation for TMJ TJR is comprehensive, beginning with thorough patient education on potential complications, such as infection, nerve injury, foreign body reactions, ankylosis, dislocation, malocclusion, persistent pain, swelling, hypersensitivity, and the need for revisions. Patients with multiple prior surgeries or chronic pain benefit from consultations with pain specialists for postoperative management, whereas those on immunosuppressants for TMJ arthritis require coordination with physicians. Imaging modalities such as Panorex, CT, and 3D reconstructions evaluate bone quality and vital structures, ensuring their suitability for prosthesis placement [11].

Patient preparation included administering anaesthesia, shaving hair to the helix top, and securing the remaining hair with tape or wraps. Skin antisepsis reduces the microbial load through preoperative showering, with broad-spectrum antibacterials applied pre-incision. Antiseptic drapes and cyanoacrylate sealants prevent bacterial migration. Prophylactic IV antibiotics, such as cephalosporins for staphylococci or vancomycin for methicillin-resistant strains, are timed within one hour of incision to minimize infection risk, with alternatives such as clindamycin for allergies [30].

Surgical execution involves superior endaural, preauricular, and posterior mandibular incisions. The superior approach dissects the zygomatic arch root, avoids the facial nerve, exposes fixation sites using condylar retractors, and isolates the condyle while protecting the internal maxillary artery. The inferior-posterior incision was placed one fingerbreadth behind the posterior border, curved anteriorly, dissecting anterior to the sternocleidomastoid and posterior to the submandibular gland to visualize the digastric tendon, incise the pterygomasseteric sling, and strip the masseter superiorly to communicate with the superior incision [31].

Condyle neck isolation uses nerve stimulators for the marginal mandibular branch and Dunn-Dautrey retractors. Soft tissue dissection medially avoids internal maxillary artery hemorrhage with retractors aiding in zygomatic and condylar preparations. A two-step condylectomy enhances safety: an initial mid-neck cut with a fissure bur removes the head and controls bleeding, followed by a higher preauricular cut. Ramus repositioning with forceps facilitated visualization. Fossa placement involves flattening the eminence with a diamond rasp, sizing for stability, and securing with 2 mm screws. Intermaxillary fixation ensures proper mating, with rasping for ramus fit and 2.7 mm screws for fixation [31,32].

Closure is layered with hemostasis, and postoperative care includes monitoring, pressure dressings for eight to 12 hours, antibiotics for seven to 10 days in high-risk cases, and prophylaxis for invasive procedures. Assessments target 30-35 mm interincisal opening, 60%-70% pain reduction, and 75% normal diet capability, with vigilant complication monitoring [31,32].

Complications

Complications in TMJ TJR, although relatively low in incidence, can significantly impact patient outcomes and require proactive management. Periprosthetic joint infection (PJI) is a rare but devastating condition, defined by criteria such as sinus tracts, pathogens in multiple samples, elevated ESR/CRP, synovial WBC counts, purulence, or histological neutrophils. Acute PJI presents with pain, fever, and discharge, while chronic PJI involves fistulae without systemic symptoms [15].

Diagnostic guidelines recommend withholding antibiotics before cultures, risk stratification, serology, and aspiration, and avoiding intraoperative Gram staining. Intrinsic complications include heterotopic bone formation, causing reduced mobility and pain, typically anterior, lateral, or posterior, without medial extension, managed with nonsteroidal anti-inflammatory drugs (NSAIDs), diphosphonates, or radiation. Dislocation, often the anterior condylar, is more common post-coronoidectomy, diagnosed clinically or via imaging, and treated with manual reduction or elastics; posterior dislocations may require device replacement [31,32].

Persistent pain after TJR stems from misdiagnosis, infection, loosening, neuroma, sensitivity, synovial entrapment, or fracture. Preventive strategies address modifiable factors, such as nutrition (serum albumin), systemic control (diabetes via HbA1c), medication adjustments (TNF blockers), smoking cessation, and preexisting infections. Intraoperative measures include antibiotic prophylaxis, eye protection, hair management, and the avoidance of device contamination. Postoperative nosocomial risks are mitigated through shorter hospitalizations and hygiene education. Overall complication rates range from 15% to 25%, with infections most amenable to treatment; however, heterotopic bone and hypersensitivity remain challenging (Table 4) [10,15,33].

Management of complications

Managing TMJ TJR complications emphasizes prevention and timely intervention to preserve function and avoid revisions. For PJI, early debridement, antibiotics, and implant retention (DAIR) are standard for acute cases, whereas chronic infections may require prosthesis removal, spacer placement, and delayed reimplantation. Prophylactic measures include perioperative antibiotics and sterile techniques, with otolaryngologist consultations for auditory canal perforations [31].

Heterotopic bone is treated through surgical excision followed by autogenous fat grafting to reduce recurrence, combined with irrigation and hemostasis. Approaches vary by location (preauricular or retromandibular), with six months of physiotherapy post-resection. Dislocations involve manual reduction under sedation with elastics for one week; recurrent cases necessitate surgical correction or custom redesign.

Nerve injuries, such as neuromas, are managed by excision if pain is localized, diagnosis via Tinel's sign, and thermal testing. Synovial entrapment arising from mechanical forces is treated with diagnostic blocks, debridement, fat grafts, and exercises. Extrinsic pain from misdiagnosis requires re-evaluation, reserving TJR for true end-stage disease, and setting realistic expectations.

Chronic pain management involves multidisciplinary approaches, including neuromodulators such as gabapentin, antidepressants, and physical therapy, to combat central sensitization. Myofascial pain is addressed through a vicious cycle or pain adaptation model that prevents the acute-to-chronic transition. Complex regional pain syndrome demands rehabilitation, psychotherapy, medications (NSAIDs, opioids), interventions (blocks, stimulation), and alternatives, such as acupuncture. Specific issues such as temporal tendinitis or coronoid impingement involve anesthetic infiltration or coronoidectomy. Frey syndrome is treated with botulinum toxin. Long-term follow-up detects wear and ensures collaborative care with pain specialists for nonsurgical resolution (Table 4) [32].

**Table 4:** Common complications and management strategies in TMJ TJR DAIR: debridement, antibiotics, and implant retention; NSAIDs: nonsteroidal anti-inflammatory drugs; TMJ: temporomandibular joint; TJR: total joint replacement

Complication	Incidence (%)	Mechanism	Prevention	Management	Prognosis	Supporting References
Periprosthetic joint infection	1–5	Intraoperative contamination or biofilm	Strict asepsis, antibiotic prophylaxis	DAIR or staged revision	Good if early	Yoda et al., 2020 [31]; Gerber et al., 2025 [15]
Heterotopic ossification	8–15	Bone proliferation around the prosthesis	NSAIDs, fat graft interposition	Surgical excision, fat graft	Moderate recurrence	Gerber et al., 2025 [15]; Sidebottom et al., 2008 [32]
Dislocation (Anterior > Posterior)	3–7	Malalignment, fixation weakness	Proper intra-op alignment	Manual reduction, revision	Generally good	Yadav et al., 2021 [13]
Facial nerve injury	2–6	Retraction or surgical trauma	Nerve monitoring, gentle dissection	Physiotherapy, neuromodulators	Often partial recovery	Eckelt et al., 1999 [26]; Gopal Krishnan et al., 2023 [27]
Hypersensitivity/Allergy	<2	Metal ion sensitivity	Pre-op allergy testing	Device replacement with an inert alloy	Variable	De Meurechy et al., 2018 [22]

Recent advances

Recent advances in TMJ TJR have focused on bioengineered solutions to overcome alloplastic limitations such as the inability to respond to biochemical cues, leading to adaptation issues. Autogenous tissues adapt but involve donor morbidity; costochondral grafts succeed owing to load-bearing cartilage, but tissue engineering (TE) offers customized alternatives [7]. TE integrates stem cells, signals, scaffolds, and perfusion to create viable replacements ex vivo [25].

Scaffolds provide biodegradable frameworks for cell attachment and remodeling, ideally matching tissue properties and promoting angiogenesis. The categories include non-bioabsorbable, bioabsorbable, and natural polymers. Dynamic interactions via mechanotransduction are key, with pore geometry influencing tissue type; 150 μm limits vascularization, while 300-563 μm optimizes bone formation [19]. Artificial scaffolds have demonstrated potential for enhancing the regulation of structural characteristics; nevertheless, supplementary procedures are frequently necessary to facilitate biomimetic cellular communication [20].

The process involves 3D imaging of condylar-ramus constructs, manufacturing via printing, cell loading or homing, implantation with fixation, and potential disc integration in stages [24]. Challenges, such as ligament technology, persist, but advances promise functional and biocompatible replacements with long-term remodeling potential.

## Conclusions

Total TMJ TJR has been established as a reliable solution for managing end-stage TMJ pathology, significantly improving patient function and quality of life. Over the decades, advancements in biomaterials, surgical techniques, and custom device design have addressed early challenges, reducing complications such as infections and heterotopic bone formation through refined protocols. The integration of CAD/CAM with wear-resistant materials, such as UHMWPE, has enhanced prosthesis longevity and performance. Despite these challenges, complications such as nerve injuries and dislocations require vigilant management, often necessitating multidisciplinary approaches. This field is poised for a transformative shift with bioengineered solutions, including bioresorbable composites and nanomaterials, which are promising for bridging alloplastic and biological reconstructions. These innovations, supported by ongoing research, hold the potential to minimize donor morbidity and optimize long-term outcomes, ensuring TMJ TJR remains a cornerstone of advanced maxillofacial care. TMJ total replacement has transitioned from salvage therapy to a reconstructive standard. As bioresponsive materials and digital simulation mature, the next decade may witness prostheses that not only restore form but also biologically participate in joint remodeling.
